# Land degradation sensitivity and desertification risk in Harrir region, northern Iraq

**DOI:** 10.1016/j.heliyon.2024.e27123

**Published:** 2024-02-24

**Authors:** Karwan Obaid Hamad, Abdulkadir Surucu

**Affiliations:** a Department of Geography, College of Arts, Salahaddin University, Erbil, Northern, Iraq; b Department of Soil Science and Plant Nutrition, Faculty of Agriculture, Harran University, Sanliurfa, Turkey

**Keywords:** Land degradation, Desertification, MEDALUS, GIS, ESI, Harrir

## Abstract

Soil degradation and desertification are persistent ecological issues that present significant challenges worldwide. An accurate evaluation of land susceptibility to desertification is essential for developing suitable strategies or policies to address it on national scales. Modified Mediterranean Desertification and Land Use (MEDALUS) model is widely utilized to assess environmental and desertification sensitivity. This study employed MEDALUS model to identify environmentally sensitive areas prone to desertification in the Harrir region, northern Iraq. A total of 102 soil samples were collected from 0 to 20 cm depth covering a land area of 279.36 km^2^. Environmental sensitivity area index (ESAI) was used to evaluate sensitivity of the study area to environmental changes. The results indicated that ∼68.18 km^2^ of the study area would be exposed to land degradation and desertification. Fragile (F) regions accounted for 39.63 km^2^, underscoring the need for effective management and conservation practices. Only a small portion of the region (2.81 km^2^) was classified as ‘critical’ (C). Further analysis revealed that fragile sub-classes F1, F2, and F3 accounted for 11.84%, 17.16%, and 14.19% respectively, while critical subclass C1, C2, and C3 areas accounted for 10.97%, 9.12%, and 1.006% respectively. The remaining areas were either classified as unaffected or had potential for being influenced by environmental changes. Approximately 24.41% of the study area had the potential for being influenced by environmental sensitivity. This highlights the importance of implementing effective management and conservation practices to protect fragile regions in the study area. Policymakers and land managers can effectively prioritize and implement targeted interventions to prevent further soil degradation and desertification in the Harrir region of northern Iraq.

## 1 Introduction

Land sensitivity refers to the ability of land to degrade or decertify in response to various natural and human activities. Food security, economic growth, and social progress on global and regional scales are negatively impacted by land sensitivity/degradation [1]. Furthermore, desertification and land degradation are serious environmental concerns that represent considerable barriers to global sustainable development [2]. Approximately 41% of earth's geographic area consists of arid areas, which are highly susceptible to desertification and land degradation [3,4]. Desertification is the conversion of productive lands into desert-like environments by human activities and climate change [5]. Arid environment [6], excessive livestock grazing [7], low vegetation cover [8], and deforestation and wrong agricultural practices [9] are some of the reasons leading to soil desertification. Desertification is a significant challenge for developing nations, and its severity is projected to increase in the future. Nevertheless, it will negatively affect food availability, water, and agricultural resources, as well as the long-term viability of natural resources [10]. Harrir region in northern Iraq is facing severe desertification/land degradation problems because of human activities and climate change [11]. Assessing the vulnerability of the landscape to human activity and climate change is an accepted method for measuring land sensitivity and desertification [12]. Understanding land sensitivity/degradation is critical for identifying vulnerable regions and developing suitable land management and conservation measures. A thorough understanding of origins, patterns, and trends of land degradation and desertification is necessary for its effective management [13].

The Mediterranean Desertification and Land Use (MEDALUS) model has been developed as a significant tool for comprehending and assessing the processes of land degradation and desertification [14]. The MEDALUS model has become more popular for performing sensitivity assessments on land degradation globally due to its greater advantages over other models. These advantages include quick results, flexibility in handling different types of data, and simple model building [15]. The MEDALUS model was developed in 1990s, and it integrates scientific study, field investigations, and expert knowledge to assess the susceptibility of certain locations to desertification [16]. It is made up of a comprehensive collection of indicators that provide a standardized framework for monitoring and measuring land degradation and desertification, with an emphasis on characteristics such as soil, climate, vegetation, and management index [17]. The MEDALUS model has been successfully used to quantify land degradation and desertification risk in numerous studies [9,15,[18], [19], [20], [21], [22], [23], [24], [25], [26], [27]].

Soil is an important indicator of land degradation and desertification because it has a direct impact on agricultural output and the functioning of ecosystems [28]. Wind and water erosion, chemical deterioration, and physical compaction can cause soil erosion and degradation [29]. However, climate is another important element influencing land degradation and desertification [30]. Changes in temperature and precipitation patterns can cause changes in vegetation distribution, change hydrological regimes, and increase erosion risk [31]. Vegetation is both an indication and a cause of land deterioration and desertification. Vegetation dynamics are tightly connected to climate and soil conditions, and changes in vegetation cover, composition, and productivity can give insight into the amount and severity of land degradation, the management index is critical in recognizing and combating land degradation and desertification [32]. It assesses the efficacy of various land management methods in minimizing and avoiding these processes [33]. This index considers factors such as agricultural methods, water management, and land-use changes, offering a complete evaluation of the human-induced influences on the landscape [34].

Furthermore, remote sensing and geographic information system (GIS) technologies have shown to be useful instruments for monitoring and mapping land degradation and desertification processes over huge regions and over long time periods [19,23,24,35]. Satellite imaging, for example, provides vast coverage and high-resolution data, allowing for the detection and characterization of land cover changes related with land degradation [[23], [24], [25],^36^]. The GIS, on the other hand, allows for the incorporation and analysis of various geographical data, making it easier to identify risk locations, contributing causes, and viable mitigation techniques [4].

The combination of MEDALUS with remote sensing and GIS enables a thorough and accurate evaluation of land degradation and desertification on a geographical and temporal scale [37]. The MEDALUS model has not been used to assess land degradation and desertification risk in Harrir region. Therefore, the current study employed MEDALUS model to investigate land degradation and desertification risk in the Harrir region. The major objective of the current study was to assess areas that are vulnerable to land degradation and desertification by using four primary indices from the original MEDALUS method (climate quality index, soil quality index, vegetation quality index, and management quality index). It was hypothesized that a significant portion of the study area would be under land degradation and desertification risk. The result will help the policymakers to develop management options for the areas under land degradation and desertification risk in the study region.

## 2 Materials and methods

### 2.1 Study area

**Fig. 1:**
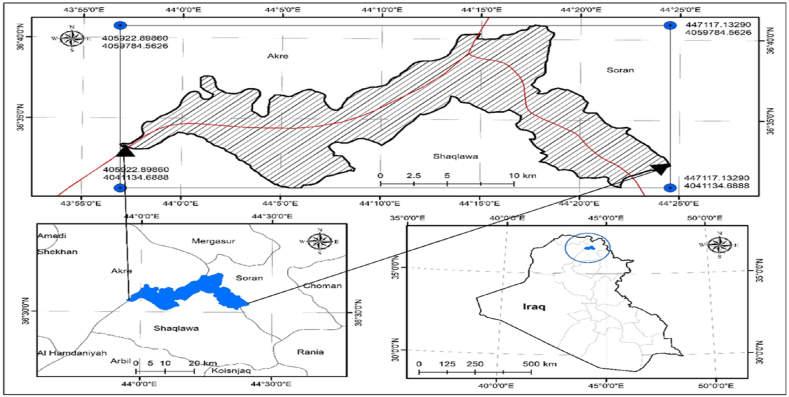
Geographic location of the study area within Iraq.

The study area (Harir district) is located in the northwestern part of Iraq in Erbil province between 36°60′00"N and 36°40′00"N latitudes, and 43°55′00"E and 44°25′020"E longitudes (Fig. 1). Harir district covers 40 villages and spans across an area of 279 km^2^. This area accounts for ∼1.8% of the total land area of Erbil Governorate. The highest point within the study area reaches approximately 1461 m above sea level, while the lowest point lies at 309 m. The area has a hot and dry summer climate and a chilly and wet winter environment. However, due to several variables including geographic location, mountain ranges, depressions, and air masses, there are variances in climatic conditions across various sections of the region. According to information from the Saqlawa station, the study area's typical annual temperatures, precipitation, and humidity are 14.5 °C, 450 mm, and 75%, respectively. The geological composition of the study area is sandstone, claystone, dolomite, calcareous and shale, gypsum, and limestone.

### 2.2 Soil sampling and laboratory analysis

**Fig. 2:**
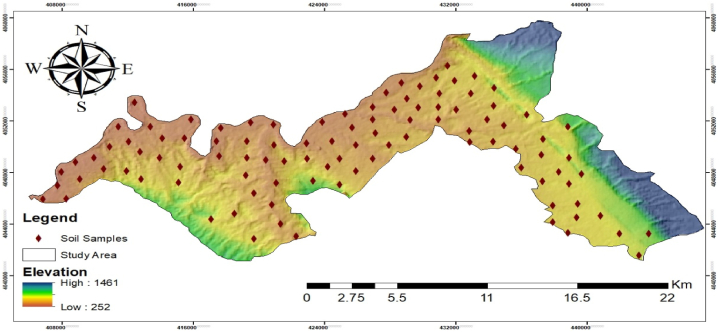
Soil sample collection points in the study area.

The study area was divided into 2 km × 2 km grids, and a total of 102 soil samples were collected from the 0–20 cm depth from the corners of each grid. Soil samples were collected with the help of soil auger, placed into plastic bags, and brought to laboratory for further analysis. Geographical coordinates were recorded to accurately record the location of each sampling point. The sampling locations are presented in Fig. 2.

Soil samples were air dried and passed through a 2 mm sieve. Afterwards, physical and chemical properties of the collected soil samples were evaluated using traditional laboratory methods. The dispersing agent used was sodium hexametaphosphate, and the hydrometer technique was employed in a sedimentation cylinder to measure particle size distribution [38]. Soil organic matter (SOM) was determined using the method described by Walkley and Black [39]. To obtain soil organic matter content, organic carbon was multiplied by a conversion ratio of 1.72 as suggested by Nelson and Sommer [40]. The electrical conductivity (EC) and soil reaction (pH) were determined in a saturated paste, following the approach outlined by Rhoades [41]. The pH meter electrode standardization was achieved using standard solutions with pH readings of 3, 7, and 10, as reported in the literature. The CaCO_3_ content was determined utilizing the calcimeter technique, as described by Allison and Moodie [42].

### 2.3 MEDALUS

The MEDALUS project demonstrates a comprehensive model that effectively measures environmental sensitivity to degradation [34]. The MEDALUS model was calibrated and validated using the defined criteria and processes stated in the original MEDALUS project. The data on management quality (MQ), soil quality (SQ), climatic quality (CQ), and vegetation quality (VQ) were collected for the study region by field surveys, remote sensing, and existing databases. Each parameter was rated on a scale of 1.0–2.0, with 1.0 and 2.0 indicating the least and the highest vulnerability to degradation, respectively. These rankings were based on expert knowledge and scientific publications (Table 1, Table 2, Table 3, Table 4).

**Table 1:** Classification scheme adopted for soil parameters to compute soil quality index.

Parameters	Description	Quality	Score	Reference
Parent material	Shale	High	1	[43]
	Gypsum, shale and claystone	Medium	1.5	
	Limestone, sandstone, dolomite, calcareous and	Low	1.7	
Slope (%)	<2	Nearly level	1	[44]
	2–6	Gentling sloping	1.2	
	6–12	Moderately sloping	1.4	
	12–18	Strongly sloping	1.6	
	18–25	Moderately steep	1.7	
	25–35	Steep	1.8	
	35–60	Very steep	1.9	
	>60	Very steep	2	
SOM (%)	>6.0	High	1	[44]
	2.1–6.0	Medium	1.2	
	2.0–1.1	Low	1.6	
	<1.0	Very low	2	
Soil texture	Loam, sandy-clay-loam, sandy-loam, loamy-sand, clay-loam	Very low	1	[43]
	Sandy-clay, silt-loam, silty-clay loam	Low	1.2	
	Silt, and clay	Medium	1.6	
	Sand	High	2	
pH	<5	Very Strong acid	1.8	[44]
	5–5.5	Strong acid	1.6	
	5.5–6	Moderate acid	1.4	
	6–6.5	Slightly acid	1.2	
	6.5–7.3	Neutral	1	
	7 0.3 - 8	Slightly alkaline	1.2	
	>8	Moderate alkali	1.5	
EC	<1.2	Good	1	[44]
	1.2–2.0	Slight	1.2	
	2.0–4.0	Medium	1.5	
	4.0–8.0	Salty	1.7	
	>8.0	Very saline	2	
CaCO_3_ (%)	<2	Very low	1.4	[44]
	2.0–4.0	Low	1.2	
	4.0–8.0	Medium	1	
	8.0–15.0	High	1.2	
	>15	Very high	1.4	

SOM = soil organic matter.

**Table 2:** Classification scheme adopted for climate parameters to compute climate quality index.

Parameter	Quality	Descriptive	Score	Reference
Precipitation	<399	Very Little Rain	2	[44]
	400–499	Little rain	1.7	
	500–699	Moderate rain	1.5	
	700–899	Very Rainy	1.3	
	>900	Too much rain	1	
FAO drought coefficient	>0.65	Hamid (rainy)	1	[45]
	0.5–0.65	Semi-arid rainy	1.2	
	0.2–0.5	Semi-arid	1.5	
	0.05–0.5	Arid	1.7	
	<0.05	Dry	2	
Aridity	<50	Very good	1	[44]
	50–75	Good	1.1	
	75–100	Moderate	1.2	
	100–125	Low	1.4	
	125–150	Very low	1.8	
	>150	Bad	2	

FAO = Food and Agriculture Organization.

**Table 3:** Classification scheme adopted for climate parameters to compute vegetation quality index.

Parameter	Quality	Descriptive	Score	Reference
Fire risk	Low	Bare land, perennial agricultural crops, Perennial agricultural crops (corn, tobacco, sunflower)	1	[34]
	Medium	Annual agricultural products (cereals, pastures), broadleaf oak (mixed), Mediterranean vegetation, lemur species	1.3	
	High	Mediterranean scrub	1.6	
	Very high	Coniferous forests (pines)	2	
Drought resistance	Very low	Mixed Mediterranean scrub/solid green forest cover, Mediterranean scrub	1	[34]
	Low	Broad-leaved trees, forest, olive	1.2	
	Medium	Perennial agricultural trees (vineyard, almonds etc.)	1.4	
	High	Perennial grasslands	1.7	
	Very high	Perennial crops, pastures	2	
Vegetation cover	High	70–10	1	[44]
	Medium	40–70	1.3	
	Low	10.0–40	1.7	
	Very low	<10	2

**Table 4:** Classification scheme adopted for management parameters to compute management quality index.

Parameters	Quality	Descriptive	Score	Reference
Crop	Low	Low land use intensity (LLUI)	1	[43]
	Moderate	Medium land use intensity (MLUI)	1.5	
	High	High land use intensity (HLUI)	2	
Pasture	Low	ASR < SSR	1	[46]
	Moderate	ASR=SSR to 1.5*SSR	1.5	
	High	ASR>1.5*SSR	2	
Natural area	Low	A/S = 0	1	[46]
	Moderate	A/S < 1	1.2	
	High	A/S = 1 or greater	2	
Mining area	Low	Adequate	1	[43]
	Moderate	Moderate	1.5	
	High	low	2	
Recreation area	Low	>1	1	[34]
	Moderate	1 to 2.5	1.5	
	High	>2.5	2	
Policy	High	Complete: >75% of the area under protection	1	[45]
	Moderate	Partial: 25–75% of the area under protection	1.5	
	Low	Incomplete:	2	

Environmentally sensitive area index (ESAI) was computed by categorizing sub-indicators inside each indicator and assigning weights. Intermediate weight values were utilized to represent relative vulnerability. Weights were calculated for each index using a mix of expert judgment and statistical analysis. Ordinary kriging [47] spatial interpolation method in ArcGIS [48]was employed to generate spatial distribution maps of each index and ESAIs. The nearest neighbor approach was employed to resample all maps to a spatial resolution of 30 m.

### 2.4 Soil quality index

Soil texture, SOM, soil pH, EC, and CaCO_3_ were determined by laboratory analyses, whereas surface slope data was obtained from the digital elevation model (DEM) using ArcGIS. A robust soil quality index (SQI) capable of facilitating a comprehensive assessment of soil quality was developed using these data. The SQI was computed by using equation (1) following Morianou et al. [49].

(1) SQI = (Slope × SOM × texture × pH × EC × CaCO_3_) ^1/6^

Here, SQI = soil quality index, SOM = soil organic matter.

The computed SQI provides a consolidated assessment of soil quality indicators, yielding a quantitative value that unambiguously depicts the soil's overall health and suitability.

### 2.5 Climate quality index (CQI)

Climate quality index (CQI) evaluates numerous climatic variables to determine the susceptibility of a region to desertification and land degradation. Climate variables, i.e., precipitation, temperature, evapotranspiration, and climate variability indicators like rainfall, aridity index, and aspect were used to quantify climatic quality. The P/PET equation, which uses the Penman-Monteith technique to compute annual potential evapotranspiration (mm) and annual precipitation (mm), was used to derive the aridity index [50]. Yearly average rainfall over the long term (2000–2015) was calculated by using daily climatic data from meteorological stations located within the study area. The ANUSPLINE method was used to simulate data on evapotranspiration and long-term annual total mean precipitation while taking site height into consideration. With the assistance of this interpolation approach, it was possible to determine the limits of the study area and compile vital data for each location where soil samples were taken. The DEM data was used to identify the aspect of the soil sampling location, which has a big impact on how land degradation processes work. Along with other pertinent qualities, the MEDALUS procedures gave this component a score. Table 2 compares the ratings given to these criteria using the two methodologies. Drought parameter was categorized as semi-arid if the value was between 0.2 and 0.5 and as arid if the value was <0.2. The semi-arid condition has a CQI coefficient of 1.5, whereas the arid situation has a coefficient of 1.7. In the same way, aridity parameter was categorized as semi-arid rainy between 0.5 and 0.65 and as rainy between 0.65 and 0.75. The semi-arid rainy condition has a CQI value of 1.2. Nevertheless, the available data does not provide the precise coefficient that is linked to the rainy condition. To calculate aridity, the average annual precipitation and reference evapotranspiration (ET_0_) were determined. The aridity index (AI) was then calculated by dividing the ET_0_ by the precipitation and multiplying it by 100. The AI represents the ratio of potential evaporation to precipitation, indicating the dryness of the climate. The calculation of drought component of CQI was typically based on various meteorological variables and indicators that assess the severity and duration of drought conditions. The CQI was computed by equation (2) [51].

(2) CQI = (Precipitation × Drought × Aridity) ^1/3^

### 2.6 Vegetation quality index (VQI)

The evaluation of vegetation quality is a crucial aspect within the MEDALUS framework, as it provides valuable information about the condition and health of the vegetation cover. The vegetation quality index (VQI) incorporates various variables related to plant cover, fire risk, erosion protection, and drought tolerance (Table 3) [34]. To generate the vegetation quality data, a combination of field observations, CORINE data, and the maximum green vegetation fraction (MGF) [52] derived from MODIS-NDVI satellite images was utilized. This approach involved analyzing 8 and 15-day periods of the satellite images to determine MGVF. Additionally, plant quality-related parameters were recorded during soil sampling. Various plant types are available in the MDALUS model with respect to fire risk and drought resilience. Based on the propensity to catch fire, the fire risk was categorized from 1 to 2 for plant species (Table 3). Every plant species was given a numerical value between 1.2 and 2 according to drought resistance (Table 3).

The normalized difference vegetation index (NDVI) is the most employed method to measure vegetation cover. It serves as an indicator of the green biomass. The NDVI was calculated using the equation (NIR-R)/(NIR + R), where NIR represents the near infrared light reflected by plants, and R represents the visible red light absorbed by vegetation. The resulting NDVI values lie within −1 to +1. Positive values indicate greater plant density, which can be observed in forests, cultivated land, and green pastures. Conversely, negative values indicate lower plant density, as seen in soil, water, and snow cover [53]. Furthermore, NDVI and CORINE land cover data were classified based on fictional vegetation land cover, and in-situ observations obtained during soil sampling to evaluate plant cover. In the original MEDALUS system, areas with >40% plant cover were assigned a score of 1.0 [34]. Given the specific climate and land use characteristics of the Harrir area, regions with 50% or higher vegetation cover were assigned a score of 1.0 due to their exceptional importance [53]. The VQI was computed by using equation (3) [51].

(3) VQI = (Fire risk × Drought resistance × vegetation cover)^1/3^

### 2.7 Management quality index (MQI)

Various factors were used to assess the management quality index (MQI) in the study area. These factors included agricultural land use intensity, pasture grazing intensity, population density, and environmental protection policies [34]. Population density was an additional parameter, while the remaining factors were already part of the original MEDALUS method (Table 4). The population displacement from remote villages and hamlets to larger villages in the Harrir region has caused the abandonment of lands, impacting soil quality. Research conducted in the northern Mediterranean mountains found that land abandonment led to an improvement in soil quality due to vegetation recovery. However, higher erosion rates were recorded when certain crops like wheat and barley were abandoned. The impact of land abandonment on land quality in the study region differs based on the specific geographical conditions. Considering the unique characteristics of study area, population density was considered vital in assessing degraded lands. Improper management of rangelands (such as overgrazing) accelerates soil erosion and land degradation. Animals are not brought to mountainous districts in the study area due to security reasons, resulting in increased grazing intensity in lowlands. Therefore, pasture grazing intensity was included as a factor in the analysis of management quality. The MQI was computed according to equation (4).

(4) MQI = (land use intensity × policy enforcement) ^½^

### 2.8 Environmental sensitivity area index (ESAI)

Environmental sensitivity area index (ESAI) is an extensive approach used to assess the environmental sensitivity of lands. The model includes four critical indicators, i.e., soil quality, climatic conditions, vegetation cover, and management quality. Each indicator is given a score, and total quality ratings for each of the four aspects are calculated. These scores are used to map and categorize ecologically sensitive locations. The study areas are grouped into unaffected (N), possibly affected (P), prone (F), and critical (C) groups (Table 5). The critical and fragile regions are further subdivided into three categories based on their relative sensitivity levels, i.e., high (3), medium (2), and low (1), to increase the accuracy of sensitivity evaluation. This detailed classification helps to develop a thorough knowledge of the areas that have a critical, fragile, prospective, or unaffected status in terms of environmental sensitivity. The ESAI was computed according to equation (5) [49].

(5) ESAI = (SQI × CQI × VQI × MQI) ^¼^

SQI = soil quality index, CQI = climate quality index, VQI = vegetation quality index, and MQI = management quality index.

**Table 5:** Environmental sensitivity area index classes and sub-classes according to model score.

Indicator	Class	Score	Quality class	Quality Subclass
**ESI**	1	<1.17	Unaffected	N
	2	1.17–1.23	Potential	P
	3	1.23–1.27	Fragile	F1
	4	1.27–1.33		F2
	5	1.33–1.38		F3
	6	1.38–1.42	Critical	C1
	7	1.42–1.53		C2
	8	>1.53		C3

## 3 Results and discussion

### 3.1 Descriptive statistics of soil properties

The SOM ranged from 0.0066 to 8.65%, whereas pH ranged from 3.73 to 7.91. Similarly, EC in the study area ranged from 2.23 to 9.86 μS cm^−1^. Sand content varied from 3.26% to 79.30%, silt content ranged between 4.98% and 58.38%, and clay content differed from 15.71% to 63.70%. Likewise, CaCO_3_ content varied from 8.35 to 13.23%. The sand and SOM contents had the highest coefficients of variation (CV), i.e., 61.10% and 74.47% respectively. Although mean pH value was suitable (7.13) some regions had quite low pH values (3.73). The low pH can negatively affect the availability of essential nutrients in the soil. The presence of a high amount of CaCO_3_ indicated that most of the soils were heavy clayey, with a mean clay content of 46.69% (Table 6).

**Table 6:** Descriptive statistics of different soil properties employed to compute soil quality index.

	Sand %	Clay %	Silt %	SOM %	CaCO_3_	pH	EC μS cm^−1^	Slope
Minimum	3.26	15.71	4.98	0.066	8.35	3.73	2.23	0.0
Maximum	79.30	63.70	58.38	8.65	13.23	7.91	9.86	28.99
Mean	18.72	46.69	34.57	2.90	10.76	7.13	4.17	5.48
Standard Deviation	12.93	9.17	8.44	2.16	1.22	0.42	1.25	4.99
Kurtosis	5.51	0.88	1.64	−0.52	−0.84	42.34	4.57	6.62
Skewness	2.01	−0.86	−0.41	0.57	0.11	−5.15	1.80	2.23
CV %	61.10	19.64	24.42	74.47	11.41	5.90	29.98	91.06

SOM = soil organic matter.

### 3.2 Soil quality index (SQI)

Approximately 37.87% of the study area was categorized as high-quality soil (Table 7). This is attributed to the presence of high SOM, low CaCO_3_ content, loamy texture, neutral pH, and flat slope. On the other hand, ∼7.40% of the area was categorized as low-sensitive class, characterized by low SOM, high CaCO_3_ content, sandy texture, and high and low soil pH. The remaining 54.71% of the study area was moderately sensitive. Medium-quality soil is further divided into five different values, ranging from medium quality 1 to medium quality 5, with respective values of 19.70%, 20.79%, 8.64%, 2.4%, and 3.17% (see Table 8).

**Table 7:** Grading of soil quality index in the study area.

Indicator	class	score	Quality class	Quality subclass	Area (km^2^)	Percentage
SQI	1	<1.13	High quality		105.77	37.86
	2	1.13–1.18	Medium quality	medium quality 1	55.04	19.70
	3	1.18–1.23		medium quality 2	58.08	20.79
	4	1.23–1.28		medium quality 3	24.13	8.64
	5	1.28–1.33		medium quality 4	6.74	2.41
	6	1.33–1.46		medium quality 5	8.86	3.17
	7	>1.46	low quality		20.67	7.40

**Fig. 3:**
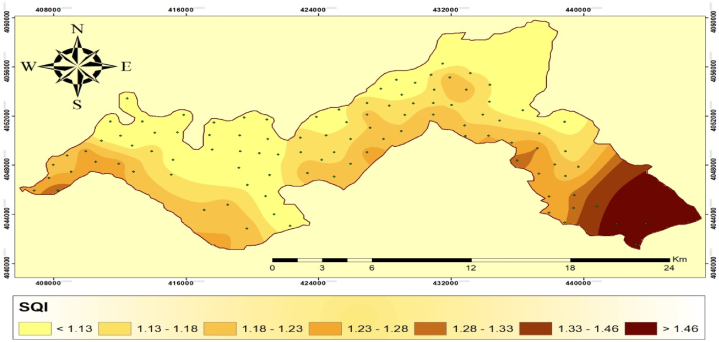
Spatial distribution map of soil quality index in the study area.

Therefore, the study concludes that a significant portion of the soil in the area is of medium quality. Spatial distribution map of SQI is given in Fig. 3. The eastern part of the study region is characterized by intensive tillage with moderate slopes, which promotes runoff and severe soil erosion. This raises concerns about water erosion. Consequently, the northern part of the region shows lower SQI values compared to the rest of the region (Fig. 3). Although there are significant areas in the southern section with deep soils, this is not the prevalent condition in most of the region. The presence of neutral pH (6.5–8.0) further contributed to the moderate SQI in this area. Overall, the study highlights the importance of the SQI as an indicator of environmental sensitivity to desertification in arid and semi-arid environments. The findings of the study emphasize the need for conservation measures in areas prone to severe soil erosion and water runoff, particularly in the south and eastern parts of the study region.

### 3.3 Climate quality index (CQI)

The high-quality CQI class covered an area of 13.82 km^2^, which accounted for 5.27% of the study region. The medium-quality class is further divided into three subclasses, each evaluated based on indicator scores ranging from 1.15 to 1.45. Subclass 1 covered 83.51 km^2^ (30.33% of the total area). Subclass 2 covered a larger footprint, i.e., 116.89 km^2^ (42.12% of the total area), whereas subclass 3 occupied 27.17 km^2^ (10.33% of total area). The low-quality class, on the other hand, covered 9.38 km^2^ (3.12% of the total research area). This suggests that only a small portion of area had unfavorable climatic conditions (Table 8). These results indicate the variability of climatic quality across the study region, with the majority falling within medium quality.

**Table 8:** Grading of climate quality index in the study area.

Indicator	Class	Score	Quality class	Quality subclass	Area (km^2^)	Percentage
CQI	1	>1.15	High quality		13.82	5.27
	2	1.15–1.25	Medium quality	medium quality 1	83.51	30.33
	3	1.25–1.35		medium quality 2	116.89	42.12
	4	1.35–1.45		medium quality 3	27.17	10.33
	5	1.45–1.56		medium quality 4	28.19	10.01
	6	>1.81	Low quality		9.38	3.12

**Fig. 4:**
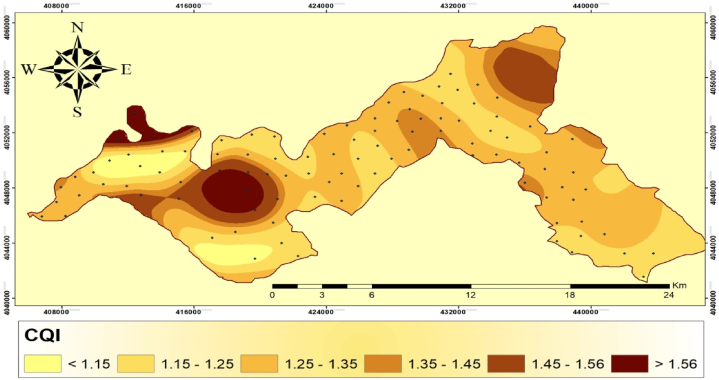
Spatial distribution map of climate quality index in the study area.

The CQI indicated land degradation vulnerability due to low precipitation (250 mm) and high evaporation (75%). The lands with higher precipitation (>400 mm) and lower evaporation (< 50% mm) had lower CQI values are regarded resistant to the degradation, and these areas were mostly located on the western part of the study area (Fig. 4). These findings offer valuable insights for policymakers and planners as the results can aid in identifying suitable locations for climate improvement initiatives. Moreover, further research and investigation are necessary to comprehend the causes and consequences of these changes in climatic quality. This information can help in devising plans to enhance overall climate resilience and sustainability of the study region.

### 3.4 Vegetation quality index (VQI)

**Fig. 5:**
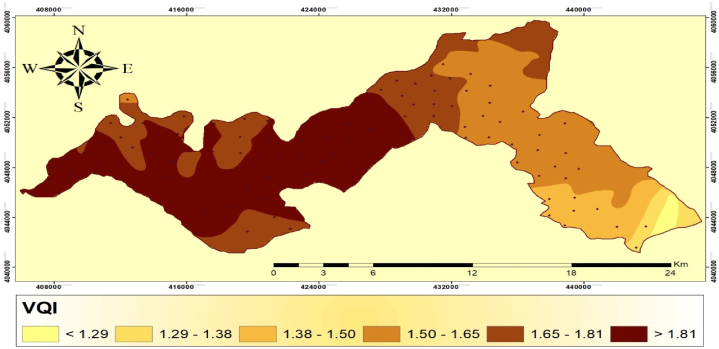
Spatial distribution map of vegetation quality index in Harri region.

The study identified six distinct VQI classes (Fig. 5). Class 1 (representing good quality vegetation) covered a small area (1.06% of the total study area) with a score <1.29. Class 2 (representing medium quality vegetation) occupied 2.44% of the study area with scores ranging from 1.29 to 1.38. Class 4 (indicating low quality vegetation) dominated 25.67% of the research area with scores ranging from 1.50 to 1.65. Subclass 5 (categorized as low-quality vegetation) covered 24.34% of the study area with scores ranging from 1.65 to 1.81. Class 6 (representing low quality vegetation) covered the largest area (38.80% of the research area) with scores >1.81.

The study revealed that most of the research area had poor vegetation quality, with classes 3–6 accounting for >96% of the total area. This highlights the urgent need for attention and management measures to prevent further deterioration and restore vegetative health. Similar to our findings, areas with low plant coverage and limited ability to withstand drought due have been identified as highly susceptible in Lesvos Island, Greece [54]. Areas of good quality vegetation (Class 1) were scarce and should be identified as critical conservation hotspots due to their higher biodiversity levels and potential ecological corridors. Medium-quality regions indicate an intermediate phase between healthy and damaged vegetation and should be regularly maintained to prevent further deterioration and potentially restore them to high-quality status. On the other hand, classes 3–6, which represent low-quality vegetation, cover a significant portion of the research region, suggesting severe degradation. Therefore, targeted management methods such as proper reforestation, erosion control, and sustainable land-use approaches should be focused on these locations. Low vegetation quality in the research area is primarily attributed to the extensive annual agricultural crops, such as wheat and barley, resulting in poor vegetation quality. In addition, the potential fire risk in wheat and barley fields during the harvest period should also be considered as a factor contributing to degradation. Although cereal fields are initially considered safe in the original MEDALUS method, the fire risk they pose should be considered [55].

### 3.5 Management quality index (MQI)

**Fig. 6:**
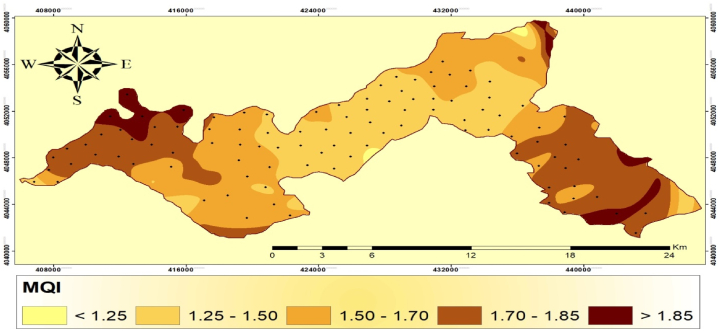
Spatial distribution map of management quality index in the Harrir region.

The MQI was determined using several indicators including population density, grazing density, farming intensity, and alternative energy sources [56] (see Table 9). The MQI was divided into five classes to assess management quality (Table 10). Class 1 represents medium quality, with a score <1.25 and covering an area of 3.040 km^2^ (2.014% of the total area). Class 2 represents medium quality, with a score ranging from 1.25 to 1.50 and covering an area of 85.37 km^2^ (30.57% of the entire area). Class 3 represents low quality, with a range of 1.50–1.70. It occurs in an isolated region, covering 190.79 km^2^ (67.4% of the total area). Moreover, low quality class is divided into three subclasses. Subclass 4 represents low quality, with a range of 1.70–1.85. It occurs in two circumstances, totaling 83.43 km^2^ (29.88% of the total land area). Subclass 5 represents low quality and has a score larger than 1.85. There are three cases within this range, covering an area of 15.82 km^2^ (5.66% of the total area) (Fig. 6).

**Table 9:** Grading of vegetation quality index in Harrir region, northern Iraq.

Indicator	Class	Score	Quality class	Quality subclass	Area (km^2^)	Percentage
VQI	1	<1.29	High		2.97	1.06
	2	1.29–1.38	Medium		6.82	2.44
	3	1.38–1.50	Low	Low quality 1	21.40	7.66
	4	1.50–1.65		Low quality 2	71.72	25.67
	5	1.65–1.81		Low quality 3	67.99	24.34
	6	>1.81		Low quality 4	108.36	38.80

**Table 10:** Grading of management quality index in the study area.

Indicator	Class	Score	Quality classes	Area (km^2^)	Percentage
MQI	1	<1.25	High quality	3.04	2.01
	2	1.25–150	Medium quality	85.37	30.57
	3	1.50–1.70	Low quality 1	91.54	31.86
	4	1.70–1.85	Low quality 2	83.43	29.88
	5	>1.85	Low quality 3	15.82	5.66

Largely, the study area mainly consisted of medium to low-quality management. Poor management quality in certain regions is primarily caused by intense traditional farming methods and overgrazing of agricultural land, particularly by sheep and goats, leading to soil compaction, reduced porosity, and increased bulk density [57]. The average population density score of 1.18 suggests that rural areas in the region have less land pressure due to a low population density. Consequently, rural regions with low populations have lower MQI values, while areas adjacent to large cities with high populations have higher MQI values due to the migration of rural residents to urban areas. Accurate data availability is vital for correctly assessing management quality, even though anthropogenic activities are the main contributors to land degradation. The accessibility of data is particularly important for evaluating the socioeconomic and environmental causes and effects of land degradation in Mediterranean nations [58]. Inadequate information, such as the extent of pasture grazing, and limited policy options make it challenging to depict land deterioration accurately. The high-quality class has the smallest coverage within the study region, providing valuable insights into the distribution and representation of different quality classes based on indicator scores.

### 3.6 Environmental sensitivity area index (ESAI)

**Fig. 7:**
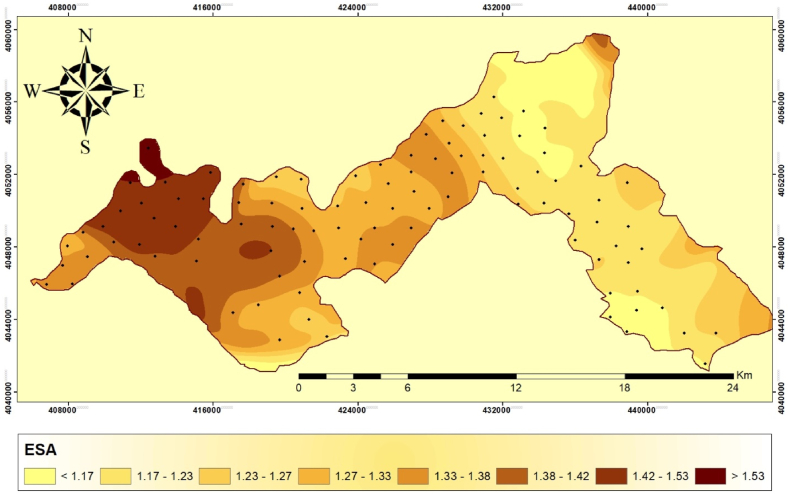
Spatial distribution map of environmental sensitivity index in the study area.

The ESAI utilizing the MEDALUS technique has provided valuable insights into the vulnerability of different regions to land degradation and desertification [[18], [19], [20], [21],^49^,^51^]. The results of the current study have been reported using a score system, which divides the locations into various groups and subclasses based on their quality. Additionally, the proportion of each class is presented to gain a better understanding of the distribution (Fig. 7).

The scoring system divides the locations into eight separate groups. Class 1 areas (with scores <1.17) are unaffected and show no signs of environmental sensitivity. Class 2 areas (making up 68.18% of the study area) could be impacted by environmental factors. They have scores ranging from 1.17 to 1.23. Class 3 and Class 4 areas (F1 and F2 subclasses respectively) have scores ranging from 1.23 to 1.27 and 1.27 to 1.33, indicating a moderate degree of sensitivity. Class 5 areas are classified as fragile (F3) and have scores between 1.33 and 1.38. These fragile places comprise 39.63% of the total area, emphasizing the need for conservation efforts. Classes 6 and 7 are critical, referred to as C1 and C2 respectively, encompass regions with scores ranging from 1.38 to 1.42 and 1.42 to 1.53. These classes indicate areas that are slightly more sensitive than the previous classes. Finally, Class 8 (identified as C3) comprises locations with scores exceeding 1.53. Fortunately, only 2.81% of the entire area falls within this highly sensitive category (Table 11). In terms of soil quality, most of the region exhibits medium characteristics. The soil demonstrates attributes such as good drainage, moderate texture, organic matter content, porosity, and bulk density. These factors contribute to the average quality of the soil in terms of water holding capacity, reduced water erosion, and a favorable environment for plant development.

**Table 11:** Grading of environmental sensitivity index in the Harrir region.

Indicator	Class	Score	Quality class	Quality subclass	Area (km^2^)	Percentage
ESI	1	<1.17	Non affected	N	31.47	11.26
	2	1.17–1.23	Potential	P	68.18	24.41
	3	1.23–1.27	Fragile	F1	33.07	11.84
	4	1.27–1.33		F2	47.93	17.16
	5	1.33–1.38		F3	39.63	14.19
	6	1.38–1.42	Critical	C1	30.65	10.97
	7	1.42–1.53		C2	25.47	9.12
	8	>1.53		C3	2.81	1.006

Consequently, the soil is considered to possess an average sensitivity to desertification. The climate of the area, however, is of poor quality due to the sensitivity of variables such as land surface temperature, aridity, and potential evapotranspiration. Vegetation quality indicators, including vertical tree density and vegetation cover, further confirm the region's vulnerability, as all constituents are classified as very sensitive or of poor quality. Grazing density and population patterns play a role in reducing the area's susceptibility to desertification. With fewer livestock grazing relative to the land mass and a lower population density, the area is less vulnerable. Previous studies by Gadzama and Ayuba [59] and Idris et al. [60] support the findings of this research. They suggest that the western portion of the study region is more susceptible to desertification, while the southern portion is relatively less vulnerable. Therefore, special consideration should be given to the northern region to mitigate the effects of desertification.

Land degradation and desertification are major issues in Iraq that are largely caused by human activity and climate change [[61], [62], [63], [64], [65], [66]]. The semi-arid environment, low rainfall, and high evaporation rates in the country are contributing to the increased desertification (39% of the country's area). This condition has negative economic effects, such as decreased agricultural productivity, increased urban migration, and slowed irrigation and construction projects [61,62]. Declining natural capacity of the soil to support crops or livestock due to poor irrigation practices, lack of water, overgrazing, and urbanization are causing desertification in the country [61,62]. Although no prior assessments on land degradation have been conducted in the study area, our findings are in line with the earlier reports indicating higher sensitivity to degradation [[61], [62], [63], [64], [65], [66]]. Reforestation, agricultural area rehabilitation, teamwork, and cutting-edge biotechnological techniques are essential to lessen the negative effects of desertification on communities, economies, ecosystems, and human health. The findings of the current study underscore the importance of effective management and conservation techniques to minimize the environmental consequences in these delicate regions.

## 4 Conclusion

The investigation carried out in Harrir region in the northern of Iraq through the integration of GIS and MEDALUS provided important information into the vulnerability of the area to land degradation and desertification. It is clear from evaluating the scores and percentages in the various classes and subclasses that the region has a variety of land qualities and uses that may be investigated. The results indicate that an extensive portion of the region is classified as fragile indicating a moderate degree of sensitivity. This demonstrates the area's potential in several fields, including urban planning, infrastructural development, and agriculture. However, it is important to highlight that a significant percentage of the land is classified as fragile or F2 suggesting a modest degree of quality and potential. This highlights the importance of proper land management and conservation techniques for preventing degradation and ensuring long-term development. In addition, a lesser fraction of the region is classified as critical or C3 signifying low quality and restricted possibilities for different activities.

These findings highlight the need of identifying and treating crucial regions through appropriate land management practices to mitigate negative consequences. Overall, the study provides significant information on land quality in northern Iraq, emphasizing the need of educated decision-making and long-term land management techniques.

## Funding

This work received no funding.

## Data availability statement

The data used to prepare the manuscripts will be available from the corresponding author on reasonable requests.

## CRediT authorship contribution statement

**Karwan Obaid Hamad:** Writing – original draft, Visualization, Software, Investigation, Formal analysis, Data curation, Conceptualization. **Abdulkadir Surucu:** Writing – review & editing, Validation, Supervision, Resources, Methodology, Conceptualization.

## Declaration of competing interest

The authors declare that they have no known competing financial interests or personal relationships that could have appeared to influence the work reported in this paper.
